# Vaccine Hesitancy Among Parents and Its Determinants During the Era of COVID-19 in Taif City, Saudi Arabia

**DOI:** 10.7759/cureus.40404

**Published:** 2023-06-14

**Authors:** Asmaa A Alzahrani, Abdulrhman N Alghamdi

**Affiliations:** 1 Preventive Medicine, Ministry of Health, Taif, SAU; 2 Family Medicine, College of Medicine, Taif University, Taif, SAU

**Keywords:** pandemic, covid-19, immunization, parents, children, pacv, vaccine hesitancy

## Abstract

Background

Vaccine hesitancy is a growing concern worldwide, particularly during the COVID-19 pandemic. The aim was to investigate vaccine hesitancy and its determinants among parents in Taif, Saudi Arabia.

Methods

A cross-sectional study was conducted among parents using a validated and reliable questionnaire, the parent attitudes about childhood vaccines (PACV). Parents of children aged (two months to seven years) attending primary health care centers (PHCC) outpatient clinics were selected using a stratified sampling technique and interviewed to fill out the pre-structured questionnaire.

Result

The study included 301 parents, with 41.2% between the ages of 30-39 years and 25.2%% between 40-49 years. Mothers constituted 69.1% of the respondents. The most common sources of information regarding vaccinations were the doctor (34.9%), the internet (27.9%), and social media (16.6%). COVID-19 influenced the beliefs of more than half (52.5%) of parents regarding the importance of vaccinations. The median PACV score for vaccination hesitancy was 23.3/100, interquartile range (IQR) (13.3-33.3). The highest hesitation was among the age group of 30-39 years old (21.6%) and those whose beliefs were not influenced by the COVID-19 pandemic (16.1% vs. 5.7%) (p-value=0.003). The study found a significant difference in vaccine hesitancy rates between the different sources of information (p-value <0.001); parents who got their information about vaccinations from social media were more likely to be hesitant about vaccinations (40%). Concerns about side effects (93.8%), thinking that vaccines are not safe (84.4%), and thinking that fewer vaccines are needed (78.1%) were the top three factors influencing vaccine hesitancy.

Conclusion

This study urges promoting vaccine uptake through healthcare providers and combating vaccine misinformation on social media. Additionally, addressing safety concerns and misconceptions about vaccine necessity, and focusing on first-time parents, younger parents, and those with lower socioeconomic status are recommended strategies to improve vaccine uptake rates.

## Introduction

Vaccine hesitancy is a growing public health concern worldwide, despite clear evidence supporting vaccine safety and effectiveness [1]. The World Health Organization (WHO) describes vaccine hesitancy as “the delay in acceptance or refusal of vaccination despite the availability of vaccination services” [2].

Vaccines have significantly reduced morbidity and mortality caused by vaccine-preventable diseases worldwide [2]. However, achieving high vaccination uptake rates is crucial to control infectious diseases effectively and prevent related complications. Childhood vaccination is a lifesaver, preventing around four million deaths worldwide each year. By 2030, measles vaccines could save 19 million lives, and Hepatitis B vaccines could save 14 million. These figures underscore the vital importance of vaccination in safeguarding public health [3].

Despite tremendous progress, the health systems have been under immense pressure due to the COVID-19 pandemic and the associated disruptions. This has resulted in 25 million children missing out on vaccinations in 2021, which is 5.9 million more than in 2019 and the highest number since 2009 [4].

According to a recent study, there was a decrease in the number of administered doses of the diphtheria-tetanus toxoid and pertussis (DTP3) and the first dose of the measles-containing vaccine (MCV1) worldwide in June 2020. The most significant decrease in the uptake of DTP3 occurred in April 2022, with a decline of 33% [5].

Vaccine-preventable disease outbreaks emphasize the need for higher vaccination coverage. In 2019, a measles outbreak in Clark County, Washington, affected 72 people, mostly children aged one to ten years, leading to a state of emergency [6]. In Italy, a low measles vaccination coverage rate of 87.3% among 24-month-old children led to a large epidemic in 2017, with over 4,885 cases reported that year [7,8].

The Kingdom of Saudi Arabia (KSA) initiated an immunization program in 1979, which initially covered vaccines for diphtheria, tetanus, pertussis, poliomyelitis, and tuberculosis [9]. The program has since been expanded to include more vaccines against other infectious diseases such as hepatitis B, measles, mumps, rubella, rotavirus, Haemophilus influenzae type b (Hib), pneumococcal disease, and meningococcal disease. Initially, getting a birth certificate was conditional on receiving certain vaccines, but those being admitted to schools must also be vaccinated. As per the Saudi Ministry of Health's immunization coverage data for 2021, the vaccination coverage for targeted diseases, such as Diphtheria, Pertussis, Tetanus (DPT), Polio, Measles, Mumps, Rubella (MMR), and Hepatitis B, was higher than 95%, which is more than the global coverage rate. Specifically, the data indicates that 97% of individuals received the MMR and DTP vaccines [10]. Nevertheless, high coverage rates do not necessarily indicate high confidence in vaccines. In 2018, the Saudi Arabian government issued an order that failure to complete vaccinations is considered neglect and mandates prosecution for those who do not comply. In some countries, the legal requirement of a complete vaccination record for a child's admission to school may influence parental decisions to immunize their children [9].

In 2018; a study in Riyadh assessed vaccine hesitancy among parents attending outpatient departments and found that 15% showed hesitancy and 34% experienced significant vaccination delays. However, only 2.5% of these delays were due to doubts about vaccine importance [11]. Another study of 300 participants, including parents, adult patients, and healthcare workers, found 17% expressed vaccine hesitancy [12].

Vaccine hesitancy is a complex and multi-faceted issue, influenced by various factors such as pre-existing beliefs about vaccines, perceptions of their benefits, attitudes towards them, past experiences with vaccines, socioeconomic status, number of children, and marital status. By understanding these factors, we can better comprehend the complex and multi-faceted nature of vaccine hesitancy and refusal among parents [13,14].

Amidst the COVID-19 pandemic, the WHO and UNICEF have warned about a concerning drop in children's vaccinations. This decline poses a serious threat as it increases the risk of preventable diseases, and the warning underscores the need to prioritize routine vaccinations to prevent further harm [15]. However, there needs to be more clarity on how the pandemic has impacted parents' hesitancy toward pediatric vaccinations in Saudi Arabia, as they are typically the primary decision-makers for their children's vaccinations. To fill this knowledge gap, this study aimed to evaluate the prevalence of vaccine hesitancy among Saudi parents in Taif City and explore how sociodemographic status and other factors contribute to parental vaccine hesitancy during the COVID-19 pandemic.

## Materials and methods

Study participant and sampling technique

This is an analytical cross-sectional interview-based targeting the parents to explore vaccine hesitancy using an electronic form of a validated and reliable questionnaire. The study was in primary health care centers (PHCC), in Taif city, Makkah province, Saudi Arabia. Parents of children aged (two months to seven years) attending PHCC outpatient clinics in Taif City were eligible for inclusion in the study. Parents of immunocompromised children, healthcare workers, and attendees for child vaccination were excluded from the sample. Due to the variability of reported hesitancy prevalence rates between the countries, a conservative hesitancy prevalence of 20% was presumed [9]. Using a precision of 5% and a confidence level of 95%, the minimum required sample of 246 was determined using EPI-INFO 7 software [Centers for Disease Control and Prevention (CDC), Atlanta, Georgia (US)]. A marginal increase of 20% was done to increase the power of the study and to account for non-respondents. The final sample size was increased to 301 subjects. To ensure the generalizability of the results, we used a stratified sampling technique. This involved dividing the total population into smaller subgroups, or strata, based on shared characteristics, and then randomly selecting participants from each group to form the final sample. In our study, stratification was based on geographic regions, Taif City was stratified into four regions (East, West, North, and South), and one primary healthcare center was chosen randomly from each of these four regions based on a list of centers obtained from the Taif health directorate. Then A systematic random sampling of every third parent was used to choose 75 participants from each center.

Data collection

The Arabic-PACV was used to measure vaccine hesitancy among parents; the Cronbach alpha for the questionnaire was 0.79, revealing good reliability [16]. PACV contains 15 items under three domains: behavior, safety and efficacy, and attitude and trust. In this study, we assessed the reliability of the Arabic-PACV questionnaire by calculating Cronbach's Alpha for all the PACV items and the items within each of the three domains. The 15 items that make up the total PACV score had a reliability coefficient of (α=0.80), indicating good internal consistency. For each of the domains, Cronbach's alpha values were 0.69 for "Behavior", 0.78 for "Safety and Efficacy", and 0.74 for "Attitude and Trust".

In addition to the PACV items, we also added sociodemographic data, beliefs about the importance of vaccinations, and parents' main source of information.

PACV-15 gives a total score of 0-30, and the total was transformed to a 100% scale by applying the simple linear transformation. The PACV total score (100%) was then dichotomized into non-hesitant parent <50, and hesitant parent ≥50 [17].

The data were collected using face-to-face interviews with parents attending PHCC. The interview started with an explanation of the study and the estimated time for completion. Parents who agreed to participate were then interviewed starting with the sociodemographic variables and other determinants of vaccine hesitancy, followed by the items of the Arabic-PACV scale.

Ethical considerations

The study received institutional ethical approval from the Institutional Review Board at King Abdelaziz City for Science and Technology (IRB registration number: HAP-02-T-067; and approval number: 541). On the participants' level, all parents were informed about the purpose of the study, and verbal consent was obtained before filling out the questionnaire. The participants' privacy was prioritized, and no personal information was collected. Participation was voluntary, and no incentives were used. All the collected data were stored securely and used for scientific purposes only.

Statistical analysis

A computer software program - SPSS (IBM Corp. Released 2021. IBM SPSS Statistics for Windows, Version 28.0. Armonk, NY: IBM Corp) was used for the data analysis. Categorical variables were presented as frequency and proportions. While continuous variables were presented using mean ± SD (standard deviation) or median and interquartile range as appropriate according to the Shapiro-Wilk test for normal distribution. The sum of the 15 items of the PACV was calculated and computed into a percentage to form the outcome variable. The outcome variable was then re-coded to be a binary variable (hesitant vs. not hesitant). The chi-square test of independence was used to test the significance with categorical variables, while the chi-square for trend was used for associations with ordinal variables. Binary logistic regression was used to calculate the group's odds ratios (OR). For significant predictors, multivariate binary logistic regression assumptions were applied, followed by forward and backward regression analyses to select the best predictors for vaccine hesitancy. The significance level was set at (0.05).

## Results

A total of (301) responses were analyzed. The age group of 30-39 years-old represented 41.2%, followed by 40-49 years old with 25.2%. Mothers were the informants for 69.1% of the responses. The majority (61.5%) indicated university as the educational level and 93.7% were married. Only 23.3% responded to the questionnaire for their first child. Of the total, only 6.6% of the parents indicated having a child with special needs or suffering from a chronic disease. See the demographic characteristics of the parents demonstrated in (Table 1).

**Table 1:** Sociodemographic characteristics of the participants (n=301) SAR: Saudi Riyals

Characteristics	Frequency	%
Parent age		
18-29	54	17.9%
30-39	124	41.2%
40-49	76	25.2%
50 & more	47	15.6%
Relationship to child		
Mother	208	69.1%
Father	93	30.9%
Educational level		
Illiterate	5	1.7%
Less than high school	21	7%
High school	58	19.3%
University	185	61.5%
Master or PhD	32	10.6%
Marital status		
Married	282	93.7%
Divorced	17	5.6%
Widowed	2	0.7%
Household income (monthly)		
< 3,500 SAR	21	7%
3,500 - 7,500 SAR	44	14.7%
7,501 - 10,000 SAR	81	27%
More than 10,000 SAR	154	51.3%
This is the first child		
Yes	70	23.3%
No	231	76.7%
Number of children in the household		
1	77	25.6%
2	59	19.6%
3	59	19.6%
4 or more	106	35.2%

The main source of information regarding vaccinations was inquired and revealed the common sources of information as follows; 34.9% from the doctor, 27.9% from the internet, and 16.6% from social media. More than half (52.5%) indicated that COVID-19 had influenced their beliefs about the importance of vaccinations. See the results regarding the source of information and COVID-19 influence in (Table 2). 

**Table 2:** Source of information and effect of COVID-19 on the importance of vaccines (n=301)

Variables	Frequency	%
Main source of information about vaccines		
Doctor	105	34.9%
Internet	84	27.9%
Social media	50	16.6%
Relatives & friends	32	10.6%
Television	16	5.3%
Written health materials	14	4.7%
Did COVID-19 disease affect your beliefs about the importance of vaccinations?		
Yes	158	52.5%
No	143	47.5%

The median PACV score for vaccination hesitancy was 23.3/100, interquartile range (IQR) (13.3-33.3). The total scores were categorized into hesitant <50/100 and not hesitant ≥50/100. The sub-categorization of the PACV score revealed 10.6% of the total was hesitant towards vaccination. Hesitancy percentages were compared across groups of demography and showed higher hesitancy among the age group of 30-39 years old, 21.8% compared to other groups. Furthermore, hesitancy showed lower odds among higher income in our sample. Significant higher odds of hesitancy were found among parents who indicated presenting to vaccinate their first child. Also, hesitancy showed significantly higher odds among parents with two children compared to four children (10.9 vs. 2.4), p-value <0.001. Those who indicated university or higher education had significantly lower odds of hesitancy, while the OR calculations among high school and lower educational levels did not show statistical significance. Those who reported that COVID-19 affected their beliefs regarding the importance of immunizations had lower odds of being hesitant (p-value=0.005). See the results detailed in (Table 3).

**Table 3:** Binary logistic regression of sociodemographic variables with vaccine hesitancy *OR: Odds ratio; SAR: Saudi Riyals

	Hesitant	Not hesitant	Beta	OR (95% CI)	p-value
Age					
18-29 years	1 (1.9%)	53 (98.1%)	-	Reference	0.003
30-39 years	27 (21.8%)	97 (78.2%)	2.69	14.8 (1.9, 111.6)	0.009
40-49 years	4 (5.3%)	72 (94.7%)	1.08	2.9 (0.3, 27.1)	0.340
50 years and more	0 (0%)	47 (100%)	-17.23	0 (0, .)	0.998
Relation to the child					
Mother	21 (10.1%)	187 (89.9%)	-0.18	0.8 (0.4, 1.8)	0.653
Father	11 (11.8%)	82 (88.2%)	-	Reference	-
Marital status					
Married	31 (11%)	251 (89%)	-	Reference	0.810
Divorced	1 (5.9%)	16 (94.1%)	-0.68	0.5 (0.7, 3.9)	0.516
Widowed	0 (0%)	2 (100%)	-19.11	0 (0, .)	0.999
Educational level					
Illiterate	0 (0%)	5 (100%)	-	Reference	<0.001
Less than high school	0 (0%)	21 (100%)	-21.2	0 (0, .)	0.999
High school	3 (5.2%)	55 (94.8%)	-21.2	0 (0, .)	0.998
University	13 (7%)	172 (93%)	-2.91	0.1 (0.01, 0.21)	<0.001
Master or PhD	16 (50%)	16 (50%)	-2.58	0.08 (0.03, 0.18)	<0.001
Household income (monthly)					
< 3,500 SAR	4 (19%)	17 (81%)	-	Reference	0.307
3,500 - 7,500 SAR	2 (4.5%)	42 (95.5%)	-0.58	1.8 (0.5, 5.9)	0.345
7,501 - 10,000 SAR	7 (8.6%)	74 (91.4%)	-1.02	0.4 (0.1, 1.6)	0.182
More than 10,000 SAR	18 (11.7%)	136 (88.3%)	-0.34	0.6 (0.3, 1.8)	0.473
This is the first child					
Yes	22 (31.4%)	48 (68.6%)	2.32	10.1 (4.5, 22.8)	<0.001
No	10 (4.3%)	221 (95.7%)	-	Reference	-
Number of children in the household					
1	23 (29.9%)	54 (70.1%)	-	Reference	<0.001
2	0 (0%)	59 (100%)	2.39	10.9 (3.6, 33)	<0.001
3	5 (8.5%)	54 (91.5%)	-17.96	0 (0, .)	0.997
4 or more	4 (3.8%)	102 (96.2%)	0.86	2.4 (0.6, 9.2)	0.214
COVID-19 affect beliefs					
Yes	9 (5.7%)	149 (94.3%)	1.16	0.32 (0.14-0.71)	0.005
No	23 (16.1%)	120 (83.9%)	-	Reference	-
Source of information					
Internet	7 (8.3%)	77 (91.7%)	-	Reference	<0.001
TV	0 (0%)	16 (100%)	18.81	0.9 (0, 0)	0.999
Social media	20 (40%)	30 (60%)	1.99	7.3 (2.8, 19.1)	<0.001
Doctor	4 (3.8%)	101 (96.2%)	-0.83	0.4 (0.1, 1.5)	0.198
Relatives and friends	1 (3.1%)	31 (96.9%)	-1.04	0.4 (0.04, 3)	0.342
Written health materials	0 (0%)	14 (100%)	-18.81	0 (0, 0)	0.999

**Figure 1:**
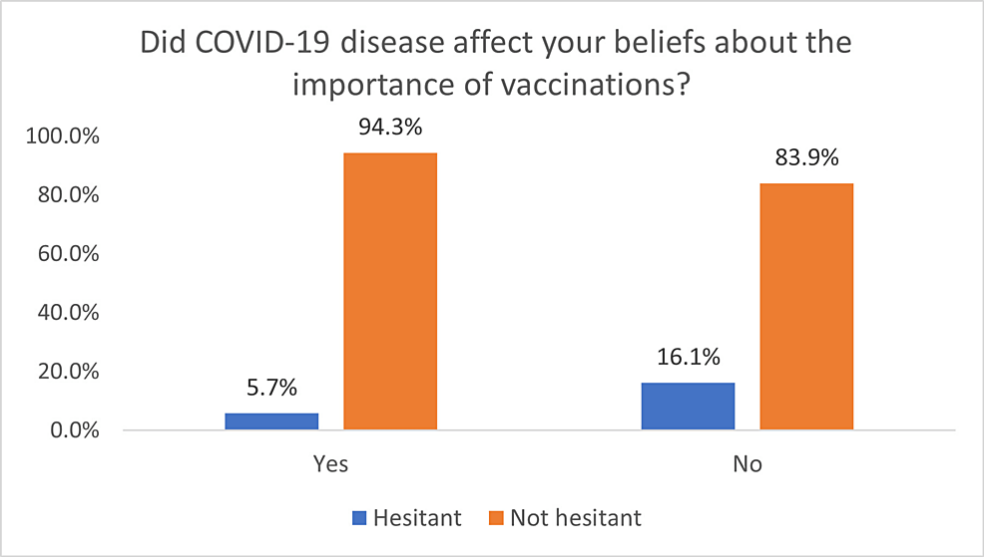
Effect of the pandemic of COVID-19 on the vaccine hesitancy OR= 0.32; p-value=0.003

**Figure 2:**
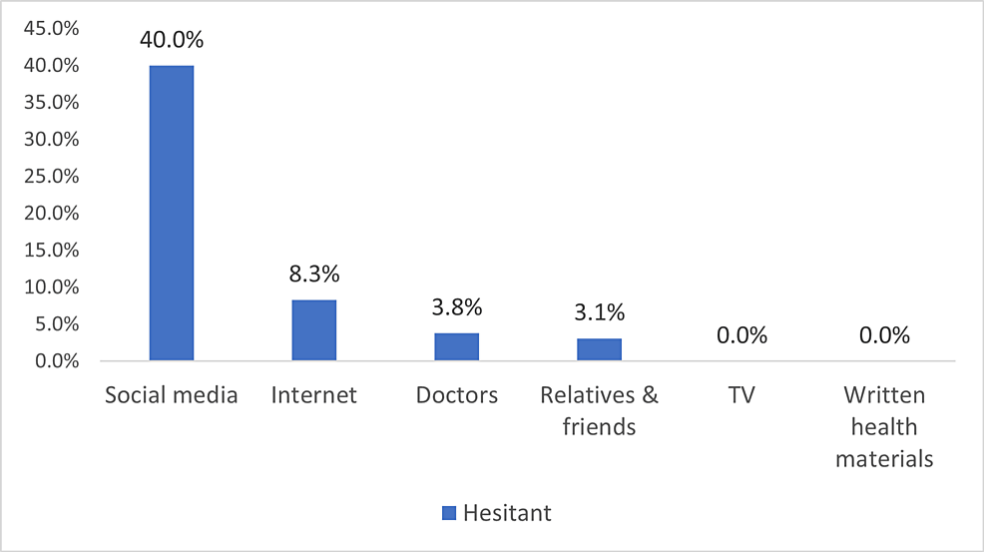
Main source of information about vaccines among the hesitant parents p-value=<0.001

The hesitancy was higher among those whose beliefs were not influenced by the COVID-19 pandemic (16.1% vs. 5.7%); this finding was statistically significant (p-value=0.003). See the results demonstrated in (Figure 1). Hesitancy was highest among parents who indicated social media as their main source of information (40%), followed by the Internet 8.3%. The differences in the hesitancy between the groups of sources of information were found to be statistical significance (p-value<0.001). See the results demonstrated in (Figure 2).

**Figure 3:**
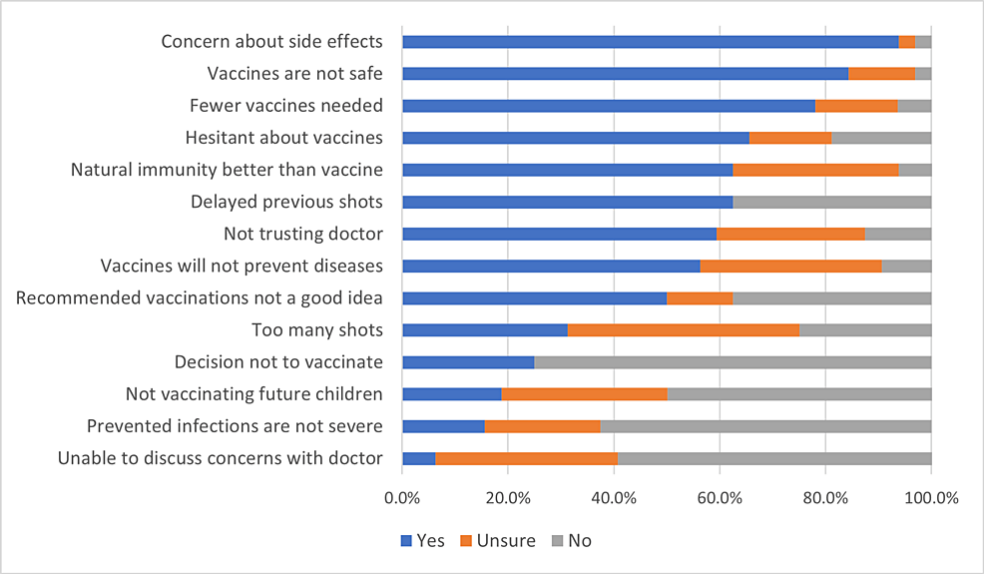
PACV factors among those who were categorized as hesitant in PACV Questionnaire PACV: Parent Attitudes about Childhood Vaccines

The factors of PACV were further investigated among those who were categorized as hesitant. Concerns about side effects were found to be the most common factor 93.8%, followed by thinking that vaccines are not safe 84.4%, followed by thinking that fewer vaccines are needed 78.1%. The lowest factors were unable to discuss concerns with the doctor 6.3% and thinking that vaccination diseases are not severe 15.6%. See the results demonstrated and detailed in (Figure 3).

A multivariate binary logistic regression model was built with two predictors: the first child and the main source of information. The model showed a prediction by Cox and Snell (R2= 20.4%). The prediction showed higher odds of hesitancy among parents presenting to vaccinate their first child and those who indicated social media as their main source of information. The aforementioned variables were significant predictors (p-value<0.001, and 0.002), respectively. See the model shown in (Table 4).

**Table 4:** Multivariate regression for first child and source of information as predictors *OR: odds ratio

Predictor	B	OR* (95% CI)	p-value
Constant	-3.11	0.04	<0.001
First child (Yes)	2.117	8.305 (3.4, 20.3)	<0.001
Source of information (internet)	Reference		<0.001
Source of information (TV)	-18.652	0 (0, .)	0.998
Source of information (social media)	1.675	5.341 (1.9, 15.1)	0.002
Source of information (doctor)	-0.994	0.37 (0.1, 1.4)	0.138
Source of information (relatives & friends)	-1.236	0.291 (0.03, 2.6)	0.27
Source of information (written health materials)	-18.712	0 (0, .)	0.999

## Discussion

Vaccines are a highly effective and safe public health measure to control disease-related morbidity and mortality [18,19]. However, a growing number of countries, regardless of their income level, are experiencing a decline in vaccine confidence which is a cause for concern [20,21]. The present study investigated the determinants of vaccine hesitancy among parents in Taif City, Saudi Arabia, during the COVID-19 pandemic. The results of the present study indicated a relatively low vaccine hesitancy rate of 10.6% among parents. These findings were much lower than previous studies in Saudi Arabia, which reported vaccine hesitancy rates ranging from 20% to 27 %. The inclusion of various sociodemographic groups in our study is a significant factor in explaining the differences between our results and those of other studies. Unlike studies that specifically target mothers or focus solely on the new COVID-19 vaccinations, our inquiry covered all childhood vaccinations [9,22,23].

Similarly, some international studies have also reported higher hesitancy rates, with approximately one-third of parents expressing hesitation to immunize their children [24,25]. The age of the children can also influence the rate of hesitancy. A previous study found a significantly higher hesitancy rate among parents with children aged 5-11 years old [26]. Altulaihi et al. reported that parental acceptance to vaccinate their children was positively associated with parental age between 31 to 40 years [22]. However, these findings were not consistent with the results of our study, which indicated that parents aged 30-39 demonstrated the highest rates of vaccine hesitancy. These findings aligned with several other studies that reported that 31-40 years of age were more hesitant than other age groups [27,28]. These results can be explained by more social media usage in young parents who can easily be influenced by fake news and misinformation about vaccines.

Furthermore, the present study found that lower-income parents were more likely to be hesitant toward vaccination. Temsah et al. reported an inverse relationship between the parent’s educational level and vaccine hesitancy [28]. These results were contrasted by Alsubaie et al., who reported that parents with higher educational levels were more vaccine-hesitant (p<0.001) [9], whereas Voo et al. did not find any correlation [29]. Similarly, previous studies from Saudi Arabia showed that education level does not influence vaccine hesitancy [12,30] study by Al-Regaiey in Riyadh, Saudi Arabia, reported no significant association between vaccine hesitancy and education level and social media usage [31].

This study also found that the main source of information regarding vaccinations was from healthcare providers, which is consistent with the recommendations of the World Health Organization (WHO) and the Centers for Disease Control and Prevention (CDC) to promote vaccine uptake [32]. The influence of healthcare providers on parental vaccination is well-established in previous studies [33,34]. Many parents reported obtaining information from the internet and social media. This pattern is consistent with previous studies that reported the internet and social media as common sources of information for parents [35,36]. The study found that parents who obtained information from social media were more likely to be hesitant towards vaccination, consistent with prior studies [35,37,38]. A cross-sectional study by Almuqbil et al. found that social media played a significant role in disseminating public anxiety among parents concerning vaccination [39]. Generally, social media has been demonstrated to negatively impact the vaccination campaign by promoting misinformation [40].

Furthermore, Saied contends that vaccine hesitancy among the general population frequently results from insufficient knowledge about vaccine safety profiles [41]. These findings were corroborated in current research as vaccine hesitancy was more prevalent among individuals whose attitudes were not shaped by the COVID-19 pandemic (16.1% vs. 5.7%). The study identified concerns about side effects, vaccine safety, and thinking that fewer vaccines are needed as the main factors contributing to vaccine hesitancy among parents. These findings are consistent with other studies that reported safety concerns and misconceptions about vaccine necessity as common reasons for vaccine hesitancy [26,28]. Safety and health-related concerns have been reported in 40-52% of vaccine hesitancy cases in Australia and Canada [42,43]. These concerns are much higher in Saudi Arabia, where only 2-5% of people cited safety concerns in prior studies [44,45].

The study also found that parents presenting to vaccinate their first child were more likely to be hesitant. These findings align with a study by Azizi et al. from Malaysia who reported that parents with their first child were four times more likely to be hesitant than others [46]. Furthermore, Napolitano et al. found that parents with their firstborns needed extra information to vaccinate their children [47].

Although the current study has several strengths, including highlighting factors that contribute to vaccine hesitancy, it also has certain limitations that should be considered while interpreting findings. The interview-based nature of the study may introduce social desirability bias. This occurs when participants provide answers they believe are socially acceptable or desirable rather than their true attitudes or behaviors. Other factors, such as cultural or religious beliefs as well as the availability, accessibility, and acceptability of vaccines, may also play a role in vaccine hesitancy among parents in this population but were not investigated in this study.

## Conclusions

In conclusion, the study provides valuable insights into the determinants of vaccine hesitancy among parents in Taif City, Saudi Arabia, during the COVID-19 pandemic. The study’s findings are consistent with other studies conducted in Saudi Arabia and worldwide, and they have important implications for policymakers and healthcare providers to address vaccine hesitancy. The study emphasizes the need to promote vaccine uptake through healthcare providers and to counter misinformation and misconceptions about vaccines on social media platforms. The study also highlights the importance of addressing safety concerns and misconceptions about vaccine necessity among parents. Additionally, the study underscores the need to focus on first-time parents, younger parents, and those with lower socioeconomic status to improve vaccine uptake rates. Future research should focus on understanding the factors underlying vaccine hesitancy and developing effective interventions to promote vaccine acceptance and improve vaccination coverage rates.
